# Potentiation Physiologically Proven

**Published:** 1893-10

**Authors:** Gustav Jaeger

**Affiliations:** Stuttgart, Germany


### POTENTIATION PHYSIOLOGICALLY PROVEN.

PROF. DR. GUSTAV JAEGER, STUTTGART, GERMANY.

[Translated by B. Fincke, M. D. Continued from page 458.]

### V. FURTHER PHYSIOLOGICAL FACTS.

I do not want once more to tire the reader with extensive statistics, but give only briefly my later experiences in this direction. In doing so I must take my two series of measurements separately, but with the preliminary remark that I have paid no attention to the appearance of *single* zero-acts, for that would have been a special exertion of attentiveness and continual interruption of the measuring process which would have jeopardized the exactness of the measurement very much. The following, therefore, relates only to the appearance of the twitchings and the great-zero and-one:

1. The duration measurements of the swallowed potencies of Kali carbonicum. There are two points to be mentioned:

(*a.*) I have already, in their former description, mentioned the appearance of cramps, and in Table I, as well as in its discussion, considered the matter, inasmuch as there I spoke of *cramp-numbers*, and said that these are especially marked in *bold* type on Table I. We can now easily read from this table at what time and where these cramps have taken place, and from this we see: for the first time cramp-numbers appear in the 13th potency, namely, 2, the 15th potency has only 1, the 17th 5, the 19th 6, the 21st 4, the 23d 7, the 25th 5, the 27th 6, the 30th 121, the 50th 11, the 100th 14, the 1,000th 11. Therefore, also, here the same phenomena as with the numbers generally, increase of the phenomena from potency to potency, but not in a regular gradation, to which is still to be added that, as said before, the intensity of the cramps increased with the height of the potency. (*b*) The maximal actions marked by the *great one* and the *great zero* belong only to the highest of the measured potencies, and again in this way: the *great one* appears only in the 30th and 50th potency, twice each time; both *great one* and *great zero* occur only at the 100th and 1,000th potency; at the 100th potency, six times *one*, twice *zero;* at the 1,000th potency, eight times *one* and twice *zero*.

2. Second series of measurements, which I instituted with the different Alkali-salts, where again two points are to be considered: (*a*) Twitchings. These were absent at the 14th potency, only at the 16th they appear at two salts (Kali iod. and Natr. carb.). To this come, at the 17th potency, Brom-natrium; at the 18th, Natr. mur. and sulph.; at the 29th, Brom-kalium and Kali phosph.; at the 21st, Natr-phosph. and Ammon-carb.; at the 22d, Natr. nitr.; at the 23d, Ammon. phosph. and Kali carb. Only one salt, *Brom-ammonium,* is at normal also in this point, even at the 30th potency there are no twitchings. If, now, a reader finds a contradiction in that I had twitchings from the twice proved Kali carbonicum, the first time at the 13th potency, the second time at the 23d, I must intimate that the numbers of the second series of investigation (inhalation) properly correspond only with the first numbers of Table I of the experiments by deglutition, with a limitation which I pointed out previously. Now, the reader will see that on Table

I no sooner than at the 19th potency the *first* number of the series is a cramp-number. Now, indeed, there remains a difference of 4 potencies. But it must be remarked that the 22d potency had not been measured; if there had been already cramps, there would remain only a difference of 3 potencies. Two points may be urged for this. *First*, there is a great difference whether a substance is *inhaled* or *swallowed*, a theme which, as I have promised before, I shall treat under production of new neuro-analytical material of another kind in a special section, and then I shall discuss extensively the differences of the two series of Kali carb. *Second*, the difference of the object and of the time about which I also spoke before. (*b*.) The maximal actions marked by the *great zero* and the *great one* appeared from none of the salts before the 30th potency, and here also only from 4 of them—from Ammon-mur. great one, from Ammon-carb. great zero, from Natr. phosph. and Kali phosph. even two *great zeros*. The question, "Why not also from Kali carb., from which at first the maximal actions appeared at the 30th potency?" is answered by their appearance at the close of the fourth minute of this duration-measurement, therefore could impossibly appear at the second measurement marked by the initial action. In the 1,000th potency the maximal action was absent, besides at the spoken-of Brom-ammonium, Natr. nitr., Natr. phosph., Natr. bromatum, Kali phosph., Brom-kalium, Ammon. mur. and phosph., and hence also only present at six salts.

To this communication of facts which appeared at the Neural Analyses two things have to be added:

1. The described co-motions are a known occurrence of practical life, and I want to emphasize only one of them which must be known to every one who was a soldier or otherwise has handled a gun: the so-called "*Mucken*," fidgeting on shooting. On discharging the gun, no other motion should be made than a slight pull with the finger, but involuntarily other muscles pull at the same time, especially those of the eyes and the head. In the more favorable case the gun is discharged—indeed mostly without hitting the mark, but when the phenomenon is developed stronger, the gun is not discharged at all, the pull of the finger was too weak to start the trigger: *this exactly corresponds to the zero-act of Neural Analysis:* also in this case one shall and will make a finger-pull for the discharge of the hand, but it will not start and usually other muscles pull at the same time—both these phenomena have also in common: (*a*) not all recruits fail in this way at the instruction of shooting, but only the more sensibly anxious ones; (*b*) everybody knows that the phenomenon is a consequence or sign of a present *nervous excitement.* In Neural Analysis we are taught in the clearest manner by those acts which do *not* fail, but produce a jump of the hand, therefore give a number. *In a decade where zero-acts occur, the numbers are smaller,* and also the mean out of the successful acts is smaller than in a decade, which shows no failures, and small numbers are incontestable symptoms of excitement, exactly as in daily life the excitement reveals itself by more rapid speaking, rapid motions of the body, more rapid breathing and pulse. Here I once more mention the co-motions in practical life. In what other manner than by increase of the velocity of motion shows itself the excitement in man and animal? In nothing else than that with the voluntary, intended motions, involuntary unintended ones of other parts of the body associate themselves—*e. g.*, when speaking, vivid play of the features, motions of the hands and arms and finally of the whole body.

2. The *starting* and *finally* the spasms which in consequence of highly attenuated substances appear with me and certainly sooner or later, stronger or weaker with all more sensible men do not lead to presuppose that one is to have a chronoscope in hand, and to let finger-pulls act upon it, but every object serves the same purpose: a gun, the trigger of which is pulled, a match-box, a cigar-box, or whatsoever is used in the same way—*e. g.*, with some corks, knitting-needles, and a long shawl-needle with large head so arranged on a drinking-glass that after a successful pull the head of the needle strikes the glass. I have manufactured an instrument which everybody can make, by means of which the same phenomena can be observed: under the influence of high potencies finger-pulls occur after which the head of the needle does strike the glass. But the most simple experiment is this: Take a firearm and watch yourself either in a looking-glass or without it. Or a torpid individual can do this also. A more sensitive person—and such are most persons of the female sex—is making the experiment, and you observe whether or not the high potencies produce the described phenomenon of "mucken." But here, in order to avoid a misunderstanding, must be accentuated that, if *no pull is exerted*, the phenomenon does not take place. If the sensibility is sufficiently developed—as *e. g.*, with me under the influence of high potencies—twitchings can also appear without any voluntary motion; but as a rule such a one is necessary for the production of the co-motions or cramps, and these then follow exactly according to the irradiation of the reflexes. This preceding fact offers to everybody who wants to convince himself—*i. e.*, on his own body—of the exciting action of the high potencies, the possibility of doing this also when he has no apparatus; and I think that it is not unfair if I express the expectation that, if one wants to judge about the matter, he at least must prove it in this wise before. Furthermore, I remark in the interest of the after-proving: Hipp's chronoscopes are to be found in every physiological and in every superior physical laboratorium. With this the zero-acts can be studied much easier than with my pocket-chronoscope, for one sees and hears quite well that the hand is drawn from its anchor, but not intercalated in the gearing. Now, the men of "imagination," or, in modern language, of "suggestion," can convince themselves that neither *arbitrariness* nor *imagination*, but only *excitement*, is capable of producing such zero-acts; furthermore, that, when excitement is present, neither imagination nor arbitrariness are able to hinder the appearance of zero-acts. This especially annihilates the hypothesis of imagination or suggestion that, in a condition in which at all zero-acts occur, the will and imagination have no power over them. They are there when we do not want them; they are absent when we want them. Finally, everybody will, with such a chronoscope, soon convince himself whether, under the influence of high potencies, the zero-acts increase or not.

I conclude this section with a very important remark: A man who *acts* upon an irritation of physical or spiritual nature *exercises* his reflex and will-organs, and lessens their conductive resistances (*Leitungswiderstände*), and now reacts fine and always finer and stronger. This is the state in which I placed myself. The *skeptic*, however, who deems himself wise not to react upon irritations, not only dulls his acting organs, by want of use, to impotence, but he increases also besides the forces of the *checking-centres* for reflexes and will-acts, and with it falls into complete impotence. A principal skeptic must be ignored principally; he is a *can-nothing*.

